# Spatial patterns of white matter hyperintensities: a systematic review

**DOI:** 10.3389/fnagi.2023.1165324

**Published:** 2023-05-11

**Authors:** Jonas Botz, Valerie Lohner, Markus D. Schirmer

**Affiliations:** ^1^Computational Neuroradiology, Department of Neuroradiology, University Hospital Bonn, Bonn, Germany; ^2^Department of Bioinformatics, Fraunhofer Institute for Algorithms and Scientific Computing (SCAI), Sankt Augustin, Germany; ^3^Cardiovascular Epidemiology of Aging, Department of Cardiology, Faculty of Medicine and University Hospital Cologne, University of Cologne, Cologne, Germany; ^4^Department of Neurology, Massachusetts General Hospital, Harvard Medical School, Boston, MA, United States

**Keywords:** white matter hyperintensity (WMH), spatial, pattern, topology, topography, magnetic resonance, MRI, systematic review

## Abstract

**Background:**

White matter hyperintensities are an important marker of cerebral small vessel disease. This disease burden is commonly described as hyperintense areas in the cerebral white matter, as seen on T2-weighted fluid attenuated inversion recovery magnetic resonance imaging data. Studies have demonstrated associations with various cognitive impairments, neurological diseases, and neuropathologies, as well as clinical and risk factors, such as age, sex, and hypertension. Due to their heterogeneous appearance in location and size, studies have started to investigate spatial distributions and patterns, beyond summarizing this cerebrovascular disease burden in a single metric–its volume. Here, we review the evidence of association of white matter hyperintensity spatial patterns with its risk factors and clinical diagnoses.

**Design/methods:**

We performed a systematic review in accordance with the Preferred Reporting Items for Systematic Reviews and Meta-Analysis (PRISMA) Statement. We used the standards for reporting vascular changes on neuroimaging criteria to construct a search string for literature search on PubMed. Studies written in English from the earliest records available until January 31st, 2023, were eligible for inclusion if they reported on spatial patterns of white matter hyperintensities of presumed vascular origin.

**Results:**

A total of 380 studies were identified by the initial literature search, of which 41 studies satisfied the inclusion criteria. These studies included cohorts based on mild cognitive impairment (15/41), Alzheimer’s disease (14/41), Dementia (5/41), Parkinson’s disease (3/41), and subjective cognitive decline (2/41). Additionally, 6 of 41 studies investigated cognitively normal, older cohorts, two of which were population-based, or other clinical findings such as acute ischemic stroke or reduced cardiac output. Cohorts ranged from 32 to 882 patients/participants [median cohort size 191.5 and 51.6% female (range: 17.9–81.3%)]. The studies included in this review have identified spatial heterogeneity of WMHs with various impairments, diseases, and pathologies as well as with sex and (cerebro)vascular risk factors.

**Conclusion:**

The results show that studying white matter hyperintensities on a more granular level might give a deeper understanding of the underlying neuropathology and their effects. This motivates further studies examining the spatial patterns of white matter hyperintensities.

## 1. Introduction

White Matter Hyperintensities (WMHs) of presumed vascular origin are a widely studied marker of cerebral small vessel disease (SVD) (Wardlaw et al., 2013). This disease burden appears hyperintense on T2-weighted Magnetic Resonance Imaging (MRI), and is often characterized on FLuid Attenuated Inversion Recovery (FLAIR) imaging.^1^ Studies have demonstrated that their prevalence and severity increase with age (Morris et al., 2009; Das et al., 2019). Moreover, it has been demonstrated that this cerebrovascular disease burden is associated with various impairments, diseases, and pathologies, such as motor (Smith et al., 2015) and mood disorders, (van Uden et al., 2015; Lohner et al., 2017) cognitive impairment (CI), (Debette and Markus, 2010; METACOHORTS Consortium, 2016; Croall et al., 2017) dementia (DEM), (Prins et al., 2004; Debette et al., 2010) and stroke (Gerdes et al., 2006; Giese et al., 2020). Additionally, the presentation of WMHs in the brain are associated with sex (Lohner et al., 2022) and clinical factors including (cerebro)vascular risk factors like hypertension (HTN) (Longstreth et al., 1996) and diabetes type 2 (DM2) (Ferguson et al., 2003). Due to its high prevalence, multiple studies have reviewed the evidence on its prevalence and modifying factors over the years (Herrmann et al., 2007; Debette and Markus, 2010; Caligiuri et al., 2015; Wardlaw et al., 2015; Frey et al., 2019; Melazzini et al., 2021).

White Matter Hyperintensities burden is heterogeneous in location and size and appears as punctate, focal, and/or confluent lesions (Fazekas et al., 1987). It is commonly characterized using semi-quantitative visual rating scales, such as Fazekas, (Fazekas et al., 1987) Manolio, (Manolio et al., 1994) or Scheltens, (Scheltens et al., 1993) or by using fully quantitative volumetric measurements based on either manual, semi-automated, or fully automated approaches. To date, however, there is no universally established methodology for quantification, as their utility depends on availability, time costs, and quality of the imaging data (Caligiuri et al., 2015). However, with the increased prevalence of fully automated and/or deep learning-enabled methodology, volumetric evaluations are becoming more prevalent (Caligiuri et al., 2015; Kuijf et al., 2019; Schirmer et al., 2019a; Lohner et al., 2022).

While most investigations tend to summarize the WMH burden as a single volumetric measure, researchers have started to acknowledge the importance of its spatial distributions to gain additional insights into the underlying neuropathology (van Veluw et al., 2022). Several studies have therefore examined the spatial patterns of WMHs in various populations. The aim of this systematic review is to give an overview of the evidence demonstrating pathological, clinical, and cerebrovascular risk factor effects on spatial patterns of WMHs in adult populations and clinical cohorts, by summarizing the increasing evidence of spatial specificity with respect to WMH burden.

## 2. Methods

This systematic review was performed using the Preferred Reporting Items for Systematic Reviews and Meta-Analysis (PRISMA) Statement (Page et al., 2021). This review was not registered and no review protocol was prepared. The associated PRISMA checklist can be found in the Supplementary Appendix.

### 2.1. Search strategy and study selection criteria

Studies have been identified by an advanced search on PubMed. The in STRIVE (Wardlaw et al., 2013) described naming conventions for WMHs were utilized as a reference to include the most prominent terms for WMHs. Additionally, we restricted our analysis to studies with MRI FLAIR data published before February 1st, 2023. Due to the lack of consensus of nomenclature for the investigation of spatial WMH burden features, the terms “pattern,” “topology,” “topography,” and “spatial” were included. The full search string is given as: (White Matter Hyperintensity OR White Matter Lesion OR White Matter Disease OR Leukoaraiosis) AND (MRI AND (FLAIR OR (Fluid Attenuated Inversion Recovery))) AND (spatial OR pattern OR topology OR topography). Only articles written in English were considered.

All abstracts were subsequently screened for eligibility. Studies were limited to human adults (>18 years) with sample sizes greater than 20 participants/patients that investigated whole-brain WMH patterns and their relation to risk factors and diseases. Studies investigating multiple sclerosis or tuberous sclerosis were not considered, following the STRIVE recommendation (Wardlaw et al., 2013). Descriptive studies without the aim to describe the association of the observed patterns were excluded for the purpose of this review. Finally, we extended our selection by examining the cited literature of the identified studies for additional relevant articles.

### 2.2. Data collection

The screening was performed by one reviewer without the use of automated tools (JB). The final decision over study inclusion was reached in consensus with a second reviewer (MS). Data were extracted using a standardized form that captured (1) disease type(s), (2) the number of patients of each disease type, (3) age, (4) sex, (5) the quantification method, and (6) spatial pattern analysis.

## 3. Results

**FIGURE 1:**
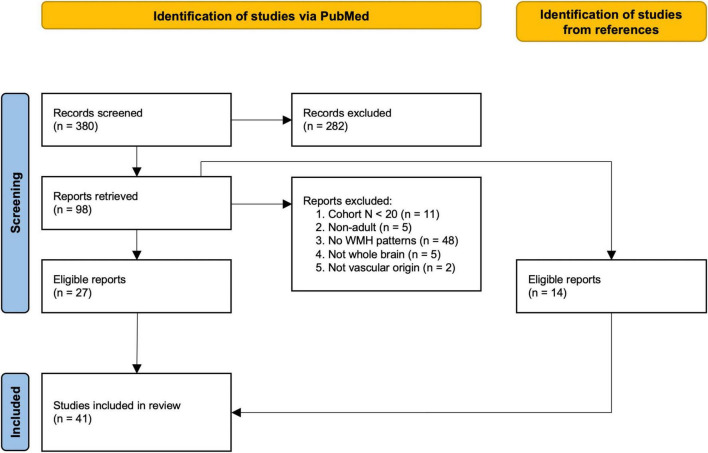
Flow-chart of study selection based on the PRISMA statement (Page et al., 2021). Studies were identified through an advanced search on PubMed. First, abstracts were screened, followed by reading the retrieved publications and excluding non-relevant studies. Exclusion criteria–Reason 1: cohort included less than 20 subjects. Reason 2: study was not performed in adult populations (>18 years). Reason 3: study did not investigate spatial patterns of WMHs (e.g., studies investigated spatial patterns of other markers, such as activation patterns of functional MRI, only included total WMH burden as a covariate, or fully descriptive studies). Reason 4: study did not assess the whole brain for analysis. Reason 5: study investigating multiple sclerosis (Kamson et al., 2012) or tuberous sclerosis (Chou et al., 2008) which do not meet the STRIVE (Wardlaw et al., 2013) definition of WMH. The reference list of each retrieved report was additionally examined for eligible studies.

A total of 380 studies were identified by the initial literature search, of which 41 studies satisfied the inclusion criteria (see Figure 1 for a detailed description of the selection phase) (Auer et al., 2001; De Groot et al., 2002; Tullberg et al., 2004; Wen and Sachdev, 2004; Yoshita et al., 2006; Saka et al., 2007; Holland et al., 2008; Stenset et al., 2008; Wen et al., 2008; Dalaker et al., 2009; Bae et al., 2010; Bunce et al., 2010; Murray et al., 2010; Jefferson et al., 2011; Kim et al., 2011; Smith et al., 2011; Rostrup et al., 2012; Biesbroek et al., 2013; McGuire et al., 2013; Ai et al., 2014; Birdsill et al., 2014; Quattrocchi et al., 2014; Brickman et al., 2015; Torso et al., 2015; Charidimou et al., 2016; Al-Janabi et al., 2018; Griffanti et al., 2018; Altermatt et al., 2019; Damulina et al., 2019; Graff-Radford et al., 2019; Schirmer et al., 2019b; Weaver et al., 2019; Wu et al., 2019; Brugulat-Serrat et al., 2020; Moscoso et al., 2020; Fang et al., 2021; Gaubert et al., 2021; Garnier-Crussard et al., 2022; Grey et al., 2022; Phuah et al., 2022; Thu et al., 2022). These studies included cohorts based on mild cognitive impairment (MCI) (15/41), Alzheimer’s disease (AD) (14/41), Dementia (DEM) (5/41), Parkinson’s disease (PD) (3/41), and subjective cognitive decline (SCD) (2/41). Six of the 41 studies investigated cognitively normal cohorts, two of which were population-based (Murray et al., 2010; Brugulat-Serrat et al., 2020). Additionally one study described the spatial WMH patterns in an acute ischemic stroke (AIS) cohort (Schirmer et al., 2019b) and one in a population with reduced cardiac output (RedCO) (Jefferson et al., 2011). Cohorts ranged from 32 to 882 patients/participants [median cohort size: 191.5, 51.6% female (range 17.9–81.3%)]. The mean age of the investigated cohorts ranged from 30 to 80 years [median: 69.9 years; mean age not reported in two studies (Quattrocchi et al., 2014; Thu et al., 2022)].

### 3.1. WMH quantification

To study spatial features of WMH burden and its association with clinical correlates and risk factors, it is necessary to characterize the extent of the burden. In the identified studies, WMHs were assessed by employing qualitative/semi-quantitative (*n* = 6), or fully quantitative approaches (*n* = 35). Most studies utilized either semi- or fully automated WMH segmentation methodology (31/35), eleven of which were based on respective in-house developed algorithms, six using the Lesion Segmentation Toolbox (LST), (Schmidt et al., 2012) and the remainder employed other available tools like the Brain Intensity AbNormality Classification Algorithm (BIANCA) (Griffanti et al., 2016) or the Medical Image Processing, Analysis, and Visualization (MIPAV) software package (Bazin et al., 2007). In five studies lesions were manually delineated.

#### 3.1.1. Semi-quantitative/qualitative

Semi-quantitative/qualitative measures represent the degree of lesional dispersion and severeness with help of visual rating scales. These scales provide a way to measure WMH burden and describe its topology without explicit lesion delineation. The used scales were the Fazekas (Fazekas et al., 1987) (and its derivatives; *n* = 5), and Scheltens (Scheltens et al., 1993) (*n* = 1) scales. The latter incorporates WMH topology in the form of local, lobar burden, while the Fazekas scale is employed for whole-brain evaluation.

#### 3.1.2. Fully quantitative

Fully quantitative approaches describe the disease burden as a volumetric measure, e.g., in cubic centimeters or milliliters. WMHs are delineated either manually, semi-, or fully automatically. Manual WMH segmentation, while often considered the gold standard in the field, is time-consuming and shows high intra- and inter-rater variability (Grimaud et al., 1996). To address this challenge, many automated assessment algorithms have been developed, (Kuijf et al., 2019), however, no universally applicable algorithm exists (Caligiuri et al., 2015).

### 3.2. Spatial pattern analyses

Based on the identified literature, we distinguish between three analysis approaches for studying spatial patterns of WMH burden: (1) Region of interest (ROI), (2) periventricular (PWMH) and deep WMH (DWMH), and (3) voxel-wise analysis. A comprehensive overview of the included studies and their findings is given in Table 1.

**TABLE 1:** Overview of the study cohorts investigating spatial WMH burden patterns and summary of their findings.

Study	Population	N	Sex (f; %)	Age mean (sd)	ME	FQN	Analysis			Findings
							ROI	P/D	v-w	
Auer et al., 2001	CADASIL sSAE	28 24	53.6 62.5	49.9 (-) 65.0 (-)	M	N	X			Increased temporal WMH in CADASIL
Tullberg et al., 2004	DEM MCI C	26 30 22	19.2 26.6 63.6	76.5 (8.7) 78.2 (7.7) 76.6 (6.5)	M	Y	X			Increase WMH in frontal lobe ->Poor executive function and episodic memory
Wen and Sachdev, 2004	C	477	47.4	62.6 (1.5)	A	Y	X			Increased PWMH across lobes associated with age ACA and MCA: higher prevalence with age
Wen et al., 2008	C	428	54.2	46.7 (1.4)	A	Y	X			Increased PWMH across lobes associated with age
Bunce et al., 2010	C	428	54.2	46.7 (1.4)	S	Y	X			Increased WMH right temporal lobe-> decreased cognitive measures
Murray et al., 2010	C	148	56.1	79.0 (5.2)	S	Y	X			Increased parietal WMH -> reduced balance and postural support
McGuire et al., 2013	NDCS C	102 91	NA NA	36.0 (6.2) 37.0 (6.0)	M	Y	X			Increased frontal and temporal WMH in pilots with NDCS
Ai et al., 2014	DEM/MCI	56	44.6	66.8 (5.4)	S	Y	X			Increased temporal WMH -> impaired cognitive function Increased parietal WMH -> decreased MoCA score
Brickman et al., 2015	AD	303	69.0	79.2 (5.3)	S	Y	X			Increased parietal WMH in possible AD
Schirmer et al., 2019b	AIS	882	38.3	65.2 (14.8)	M	Y	X			ACA: higher prevalence with age, small vessel stroke, HT, smoking ACA: lower prevalence with HLD MCA: lower prevalence with age PCA: higher prevalence for male sex PCA: lower prevalence with age, small vessel stroke
Wu et al., 2019	MCI C	22 98	59.0 64.0	70.0 (8.5) 69.9 (8.7)	A	Y	X			Increased bilaterally WMH in inferior-occipital white matter in MCI
Brugulat-Serrat et al., 2020	C	561	61.0	57.4 (7.5)	A	Y	X			Increased DWMH -> poor executive function Increased PWMH -> poor episodic memory
Fang et al., 2021	PD C	21 33	47.4 45.5	67.6 (9.2) 72.5 (10.6)	M	Y	X			Increased WMH in frontal lobes -> impaired motor function
Garnier-Crussard et al., 2022	AD C	54 40	40.7 50.0	71.0 (9.1) 69.7 (6.9)	A	Y	X			Increased WMH in posterior regions (parietal, temporal, occipital) associated with AD
Thu et al., 2022	tAD LPA-AD PCatro-AD	50 75 39	58.0 58.7 61.5	- (Med: 63) - (Med: 68) - (Med: 64)	A	Y	X			Increased WMH in left parietal lobe in LPA-AD compared to PCatro-AD Increased WMH in right occipital, parietal and temporal lobes in PCatro-AD compared to LPA-AD Increased WMH in occipital lobe in LPA-AD and PCatro-AD compared to tAD
Holland et al., 2008	AD/MCI CAA C	41 32 29	48.8 62.5 62.1	71.2 (5.1) 75.1 (7.1) 72.3 (7.1)	S	Y			X	Increased PWMH associated with age Increased frontal WMH associated with age Increased WMH in regions with lower relative perfusion
Dalaker et al., 2009	PD C	163 102	39.3 54.9	65.7 (9.4) 66.2 (9.1)	S	Y			X	No regional effects in PD patients
Jefferson et al., 2011	RedCO	32	37.5	72.0 (8.0)	S	Y			X	Increased WMH in regions with lower normative perfusion
Smith et al., 2011	DEM MCI C	11 96 40	45.0 61.0 65.0	71.2 (4.7) 72.9 (5.5) 74.1 (6.9)	S	Y			X	Increased PWMH -> poor executive function andpoor episodic memory
Rostrup et al., 2012	C	605	53.4	74.1 (5.1)	S	Y			X	Increased PWMH -> increase with age, male, smoking, alcohol consumption Increased DWMH -> HT
Quattrocchi et al., 2014	BM w/LC BM w/out LC	107 93	29.0 40.9	- (Med: 63) - (Med: 60)	S	Y			X	Inverse relationship between WMH and BM Increased WMH in occipital and frontal lobes in patients with LC
Torso et al., 2015	MCI C	31 26	54.8 57.7	67.5 (7.0) 71.3 (8.1)	S	Y			X	Bilateral WMH in anterior thalamic radiation associated with NPI-12 apathy score
Al-Janabi et al., 2018	MCI C	36 26	47.2 65.4	73.5 (8.0) 76.8 (6.1)	S	Y			X	Increased posterior WMH -> lower CSF amyloid β Increased DWMH -> HT
Altermatt et al., 2019	CI	878	58.7	68.2 (7.6)	S	Y			X	No regional association with cognitive performance
Damulina et al., 2019	AD C	130 130	63.1 61.5	73.8 (5.3) 74.6 (7.0)	S	Y			X	Increased PWMH in AD
Graff-Radford et al., 2019	AD	424	45.0	75.0 (8.4)	A	Y			X	Increased amyloid burden associated with local WMH associated with lobar CMBs
Moscoso et al., 2020	AD	190	46.8	73.1 (6.3)	S	Y			X	Increased frontal and parietal WMH -> faster rates of amyloid accumulation
Phuah et al., 2022	MCI DEM C	529 166 346	47.7	72.9 (7.6)	A	Y			X	Increased deep frontal WMH -> HT and DM2 Increased juxtacortical WMH -> CAA, male, CI
De Groot et al., 2002	C	563	49.9	73.5 (10.6)	M	N		X		Increased PWMH -> increased rate of cognitive decline
Saka et al., 2007	AD MCI	25 17	44.0 29.4	68.9 (5.8) 74.7 (6.4)	M	N		X		Increased PWMH in AD Increased PWMH -> increased interuncal distance
Stenset et al., 2008	MCI	217	50.2	69.7 (8.9)	M	N		X		Increased DWMH -> decreased Mini Mental Status Examination
Griffanti et al., 2016	C	563	17.9	69.6 (5.3)	A	Y		X		Increased PMWH -> impaired cognitive function
Charidimou et al., 2016	CAA-ICH HA-ICH	319 137	50.5 43.1	73.9 (1.8) 67.2 (3.5)	M	N		X		Increased DWMH in CAA Increased PWMH in HA
Grey et al., 2022	AD + MCI PD + PDMCI C	40 42 55	70.0 29.0 69.0	71.5 (7.1) 63.8 (9.7) 67.0 (7.3)	M	Y		X		Increased PWMH in PD and PDMCI Increased DWMH with cognitive performance
Bae et al., 2010	PDis C	24 24	46.8 50.0	32.3 (6.6) 30.1 (6.1)	M	N	X	X		Increased DWMH in PDis Increased frontal WMH in PDis
Kim et al., 2011	AD MCI C	37 23 22	73.0 47.8 72.7	70.5 (7.0) 66.5 (7.2) 67.6 (6.2)	S	Y	X	X		Increased PWMH -> impaired cognitive function Increased parieto-occipital WMH -> decreased neuropsychological scores
Yoshita et al., 2006	AD MCI C	26 28 33	61.5 39.3 69.7	73.4 (8.1) 74.8 (8.2) 79.6 (6.8)	S	Y	X		X	Increased WMH in CC -> increase in CI Increased WMH in periventricular ROIs -> HT
Biesbroek et al., 2013	artd	516	18.0	56.7 (9.4)	S	Y	X		X	Increased WMH in SLF and ATR -> poor executive functioning
Birdsill et al., 2014	AD	349	68.2	59.7 (6.4)	A	Y	X		X	Increased WMH in CC, CR and right cingulum -> reduced speed and flexibility
Weaver et al., 2019	AD DEM MCI SCD	97 37 118 121	52.7 39.4 39.8 46.3	68.5 (7.6) 66.4 (6.7) 68.0 (7.4) 63.5 (7.5)	A	Y	X		X	Increased bilateral parieto-occipital periventricular WMH -> lower CSF Aβ
Gaubert et al., 2021	AD MCI SCD C	25 51 28 51	64.0 56.9 60.7 47.1	68.0 (10.2) 73.3 (7.2) 67.1 (7.4) 70.8 (6.4)	A	Y	X		X	Increased WMH in CC -> increased AV45-SUVR Increased WMH in temporo-parietal region ->decreased FGD-SUVR

A, automatic; ACA, anterior cerebral artery; AD, Alzheimer’s disease; AIS, acute ischemic stroke; artd, arterial disease; ATR, anterior thalamic radiation; AV45, florbetapir; BM, brain metastases; C, control; CAA, cerebral amyloid angiopathy; CADASIL, cerebral autosomal dominant arteriopathy with subcortical infarcts and leukoencephalopathy; CC, corpus callosum; CI, cognitive impairment; CMBs, cerebral microbleeds; CR, corona radiata; CSF, cerebrospinal fluid; DEM, dementia; DM2, diabetes mellitus type 2; f, female; FDG, fluordesoxyglucose; FQN, fully quantitative; HA, hypertensive arteriopathy; HLD, hyperlipidemia; HTN, hypertension; ICH, intracerebral hemorrhage; LC, lung cancer; LPA-AD, logopenic progressive aphasia; M, manual; MCA, middle cerebral artery; MCI, mild cognitive impairment; ME, method; MoCA, Montreal cognitive assessment; N, cohort size; NDCS, neurologic decompression sickness; NPI, neuropsychiatric inventory; P/D, PWMH/DWMH; PCA, posterior cerebral artery; PCatro-AD, posterior cortical atrophy; PD, Parkinson disease; PDis, panic disorder; RedCO, reduced cardiac output; ROI, region of interest; S, semi-automatic; SCD, subjective cognitive decline; sd, standard deviation; SLF, superior longitudinal fasciculus; sSAE, sporadic subcortical arteriosclerotic encephalopathy; SUVR, standardized uptake value ratio; tAD, typical amnestic Alzheimer’s Disease; v-w, voxel-wise.

#### 3.2.1. Regions of interest

In this approach, the brain is subdivided into ROIs, based on brain areas that individual studies hypothesize to be associated with specific biomarkers. These ROIs may represent, e.g., cortical lobes, (Auer et al., 2001; Wen and Sachdev, 2004; Wen et al., 2008; Bunce et al., 2010; Murray et al., 2010; McGuire et al., 2013; Ai et al., 2014; Brickman et al., 2015; Wu et al., 2019; Fang et al., 2021; Garnier-Crussard et al., 2022; Thu et al., 2022) or other specific regions that may be relevant to the patient cohort or disease under investigation, e.g., using vascular territories (Wen and Sachdev, 2004; Schirmer et al., 2019b). To identify and define ROIs, studies often rely on image registration to a brain template, which is often derived for the general population or a control cohort, and on which the ROIs are defined.

We identified eleven studies using ROI analyses (Auer et al., 2001; Tullberg et al., 2004; Wen and Sachdev, 2004; Wen et al., 2008; Bunce et al., 2010; Murray et al., 2010; McGuire et al., 2013; Ai et al., 2014; Brickman et al., 2015; Schirmer et al., 2019b; Wu et al., 2019; Brugulat-Serrat et al., 2020; Fang et al., 2021; Garnier-Crussard et al., 2022; Thu et al., 2022). Of these studies eight utilized the definition of cortical lobes or a modified version of them as ROIs. While increased parietal WHM burden was associated with AD, (Brickman et al., 2015) reduced balance and postural support, (Murray et al., 2010) and with poor cognitive function, measured with the Montreal cognitive assessment (MoCA), (Ai et al., 2014) increased WMH burden in the frontal lobe was related to poor executive function and episodic memory (Tullberg et al., 2004) in patients with PD (Fang et al., 2021) and neurological decompression sickness (NDCS) (McGuire et al., 2013). Increased temporal WMH burden corresponded with decreased cognitive measures, (Bunce et al., 2010) and impaired cognitive function, (Ai et al., 2014) while another study associated increased inferior-occipital WMH burden with MCI (Wu et al., 2019). Additionally, Garnier-Crussard et al. (2022) and Thu et al. (2022) found WMH in the posterior regions (parietal, temporal, and occipital) to be associated predominantly with AD. Thu et al. (2022) further compared the local WMH burden across atypical variants of AD logopenic progressive aphasia (LPA-AD), posterior cortical atrophy (PCatro-AD) and typical amnestic AD (tAD). Patients with LPA-AD compared to PCatro-AD showed a higher left/right posterior lobar WMH burden, and patients with either atypical variants had more WMHs in the occipital lobes compared to patients with tAD. In patients with cerebral autosomal dominant arteriopathy with subcortical infarcts and leukoencephalopathy (CADASIL), Auer et al. (2001) found an increased WMH burden in the temporal lobes, and in those with NDCS, McGuire et al. (2013) described an increased WMH burden in the frontal and temporal lobes. Schirmer et al. (2019b) demonstrated a shift of predominance of WMH burden in a cohort of AIS patients and by using areas of cortical blood flow, i.e., vascular territories, that were associated with age, sex, small vessel stroke, hypertension, smoking, and hyperlipidemia. Similarly, Wen and Sachdev (2004) introduced cortical blood flow regions in addition to lobar ROIs. They found increased WMH burden in the anterior and middle cerebral artery regions as well as in the periventricular regions. The association between advanced age and increased periventricular WMH burden was confirmed by a follow-up study (Wen et al., 2008) of younger patients (mean age under 50 years). Finally, using a bullseye representation, Tullberg et al. (2004) showed an increased WMH burden in the deep white matter which was associated with poor executive function whereas increased WMH burden close to the ventricles was found to be associated with poor episodic memory. While this approach roughly relates to P/DWMH, discussed in the next section, they did not follow the common definitions for stratification in their work, resulting in 36 unique ROIs.

#### 3.2.2. PWMH and DWMH

Periventricular White Matter Hyperintensities and DWMH are umbrella terms for multiple definitions in the literature, all relating WMH burden to the distance with respect to the ventricular surface (Griffanti et al., 2018). In general, PWMHs are adjacent or in close proximity to the lateral ventricles, while DWMHs are located at larger distances inside the subcortical white matter. While PWMH and DWMH can be considered ROIs, this definition aims to reflect different functional, histopathological, and etiological features of WMH burden.

We identified six studies distinguishing PWMH and DWMH (De Groot et al., 2002; Saka et al., 2007; Stenset et al., 2008; Charidimou et al., 2016; Griffanti et al., 2018; Grey et al., 2022). Increased PWMH burden was found to be associated with PD, (Grey et al., 2022) AD and brain atrophy, (Saka et al., 2007) faster progression in cognitive decline, (De Groot et al., 2002) and impaired cognitive function (Griffanti et al., 2018). Increased DWMH burden was associated with a decreased mini mental status examination score in patients with MCI (Stenset et al., 2008). Another study showed increased DWMH burden in subjects with intracerebral hemorrhage and cerebral amyloid angiopathy (CAA), while subjects with intracerebral hemorrhage and hypertensive arteriopathy (HA) presented with increased PWMH burden (Charidimou et al., 2016).

#### 3.2.3. Voxel-wise

In the voxel-wise approach, each white matter voxel is studied for the presence of WMHs. After image registration to a template, the voxel-based frequency can be used to either derive maps showing clusters of WMHs within a specific cohort or serve as the basis for voxel-based lesion-symptom mapping. The advantage of voxel-wise analysis lies in the hypothesis-free, data-driven localization of lesion clusters compared to ROI approaches. Furthermore, it enables studies to combine this analysis with studying associations with other localized cerebrovascular information, such as presence of cerebral microbleeds and perfusion.

Thirteen studies utilized a voxel-wise analysis approach (Holland et al., 2008; Dalaker et al., 2009; Jefferson et al., 2011; Smith et al., 2011; Rostrup et al., 2012; Quattrocchi et al., 2014; Torso et al., 2015; Al-Janabi et al., 2018; Altermatt et al., 2019; Damulina et al., 2019; Graff-Radford et al., 2019; Moscoso et al., 2020; Phuah et al., 2022). In patients with AD and MCI, local WMH burden in the frontal and parietal lobes close to the ventricles was associated with an increased amyloid burden (Al-Janabi et al., 2018; Graff-Radford et al., 2019; Moscoso et al., 2020; Phuah et al., 2022). Increased WMH burden in voxels around the ventricles was found to be associated with advanced age, (Rostrup et al., 2012) poor executive function and episodic memory, (Smith et al., 2011) and with AD (Damulina et al., 2019). Further, juxtacortical WMH load was found to be associated with CI, male sex, and CAA, and deep frontal WMH burden with hypertension and DM2 (Phuah et al., 2022). Quattrocchi et al. (2014) showed that patients with lung cancer (LC) with brain metastases (BM) presented with increased WMH burden in the frontal and occipital lobes, compared to those in a control group. They also identified an inverse relationship between the presence of BM and WMH burden. Two additional studies measured local perfusion and demonstrated an inverse relationship between WMH burden and perfusion, meaning higher WMH burden related to low relative perfusion in the normal appearing white matter (Holland et al., 2008; Jefferson et al., 2011). In another study increased WMH burden bilaterally in the anterior thalamic radiation was found to be associated with apathy (Torso et al., 2015). Lastly, two studies reported no regional associations between WMH burden and cognitive performance (Altermatt et al., 2019) or PD (Dalaker et al., 2009).

#### 3.2.4. Mixed approach

Seven studies utilized a mixed spatial analysis approach, two with ROI as well as distinguishing between PWMH and DWMH, (Bae et al., 2010; Kim et al., 2011) and five studies applying ROI and voxel-wise analyses (Yoshita et al., 2006; Biesbroek et al., 2013; Birdsill et al., 2014; Weaver et al., 2019; Gaubert et al., 2021). Kim et al. (2011) found increased PWMH burden associated with impaired cognitive function and increased parieto-occipital WMH burden associated with decreased neuropsychological scores. Kim et al. (2011) found increased frontal and deep WMH burden in patients with panic disorder compared to controls. Four of the studies using ROI and voxel-wise analyses found local associations between WMH burden and lower cerebrospinal fluid (CSF) amyloid burden, (Weaver et al., 2019) poor executive function, (Biesbroek et al., 2013) increased vascular risk factors and CI, (Yoshita et al., 2006) and reduced speed and flexibility of executive function. Birdsill et al. (2014) and Gaubert et al. (2021) demonstrated associations of regional WMH distributions in multiple locations, such as the posterior lobes and the corpus callosum, with multimodal brain biomarkers of AD.

### 3.3. Spatial effects of vascular risk factors and clinical correlates

Some of the discussed studies reported spatial effects of vascular risk factors and clinical correlates, such as age, sex, HTN, smoking, alcohol, stroke, hyperlipidemia (HLD), and specific neurodegenerative and other diseases.

Advanced age was associated with increased PWMH (Wen and Sachdev, 2004; Holland et al., 2008; Stenset et al., 2008; Wen et al., 2008; Griffanti et al., 2018) and DWMH (Stenset et al., 2008; Wu et al., 2019) burden as well as with increased WMH burden in the parietal lobes (Holland et al., 2008). Furthermore–using vascular territories as ROIs–an association was found between advanced age and increased and decreased relative WMH burden in the anterior cerebral artery (ACA) and middle cerebral artery (MCA) territory, respectively (Wen and Sachdev, 2004; Schirmer et al., 2019b). Men showed increased PWMH (Rostrup et al., 2012) and WMH burden in the posterior cerebral artery (PCA) region, (Schirmer et al., 2019b) while women presented with increased frontal WMH burden (Bunce et al., 2010). Increased DWMH (Yoshita et al., 2006; Rostrup et al., 2012; Al-Janabi et al., 2018) and ACA territory burden (Schirmer et al., 2019b) were associated with HTN. Smoking (Schirmer et al., 2019b) and alcohol consumption (Rostrup et al., 2012) showed increased PWMH burden, while smoking also showed increased WMH burden in the ACA territory (Schirmer et al., 2019b). Patients with small vessel stroke presented with increased WMH burden in the ACA and decreased WMH burden in the PCA territory, while HLD led to a decreased WMH burden in the ACA territory (Schirmer et al., 2019b). Patients with AD showed a broad spectrum of spatial effects: they had increased WMH burden in the frontal (Tullberg et al., 2004; Graff-Radford et al., 2019; Moscoso et al., 2020) and parietal (Tullberg et al., 2004; Brickman et al., 2015; Graff-Radford et al., 2019; Moscoso et al., 2020) lobes, in the posterior cerebrum,(Gaubert et al., 2021; Garnier-Crussard et al., 2022; Thu et al., 2022) the corpus callosum, (Yoshita et al., 2006; Birdsill et al., 2014; Gaubert et al., 2021) the corona radiata, (Birdsill et al., 2014; Gaubert et al., 2021) the anterior thalamic radiation (Birdsill et al., 2014; Gaubert et al., 2021) as well as increased PWMH (Yoshita et al., 2006; Saka et al., 2007; Kim et al., 2011; Damulina et al., 2019; Graff-Radford et al., 2019; Weaver et al., 2019) and DWMH (Stenset et al., 2008) burden. Patients with MCI showed similar distributions with increased WMH burden in the parietal lobe, (Ai et al., 2014) the posterior cerebrum (Al-Janabi et al., 2018) and increased PWMH burden, (Yoshita et al., 2006; Smith et al., 2011) but also increased WMH burden in the temporal lobe, (Ai et al., 2014) which was also found to be associated with CADASIL (Auer et al., 2001). Patients with PD presented with increased WMH burden in the frontal lobe (Fang et al., 2021). Other diseases like arterial disease showed increased WMH burden in the superior longitudinal fasciculus and the anterior thalamic radiation, (Biesbroek et al., 2013) panic disorder was associated with increased DWMH burden as well as with increased WMH burden in the frontal lobe (Bae et al., 2010). NDCS (McGuire et al., 2013) and LC with BM (Quattrocchi et al., 2014) were associated with WMH burden in the frontal lobe. CAA and HA were associated with increased DWMH and PWMH burden, respectively (Charidimou et al., 2016). The associations are summarized in Table 2.

**TABLE 2:** Vascular risk factors and clinical correlates affecting spatial patterns.

Vascular risk factors and clinical correlates	Spatial effect (# studies)
Age	Increased PWMH (*n* = 5) (Wen and Sachdev, 2004; Holland et al., 2008; Stenset et al., 2008; Wen et al., 2008; Griffanti et al., 2018)
	Increased DWMH (*n* = 2) (Stenset et al., 2008; Wu et al., 2019)
	Increased parietal WMH (*n* = 1) (Holland et al., 2008)
	Increased WMH in ACA (*n* = 2) (Wen and Sachdev, 2004; Schirmer et al., 2019b)
	Decreased WMH in MCA (*n* = 2) (Wen and Sachdev, 2004; Schirmer et al., 2019b)
Sex	Increased PWMH [male (Rostrup et al., 2012; Phuah et al., 2022)] (*n* = 2)
	Increased frontal WMH [female (Bunce et al., 2010)] (*n* = 1)
	Increased WMH in PCA [male (Schirmer et al., 2019b)] (*n* = 1)
Vascular risk factors	Increased DWMH [HTN (Yoshita et al., 2006; Rostrup et al., 2012; Al-Janabi et al., 2018)] (*n* = 3)
	Increased PWMH [smoking (Schirmer et al., 2019b), alcohol (Rostrup et al., 2012)] (*n* = 2)
	Increased relative WMH in ACA (HTN, smoking, stroke) (Schirmer et al., 2019b) (*n* = 1)
	Decreased relative WMH in ACA [HLD (Schirmer et al., 2019b)] (*n* = 1)
	Decreased relative WMH in PCA [stroke (Schirmer et al., 2019b)] (*n* = 1)
Neurodegenerative diseases	Increased frontal WMH [AD (Tullberg et al., 2004; Graff-Radford et al., 2019; Moscoso et al., 2020), PD (Fang et al., 2021)] (*n* = 4)
	Increased temporal WMH [MCI (Ai et al., 2014), CADASIL (Auer et al., 2001)] (*n* = 2)
	Increased parietal WMH [AD (Tullberg et al., 2004; Brickman et al., 2015; Graff-Radford et al., 2019; Moscoso et al., 2020), MCI^64^ (Ai et al., 2014)] (*n* = 5)
	Increased posterior WMH [AD (Gaubert et al., 2021; Garnier-Crussard et al., 2022; Thu et al., 2022), MCI (Al-Janabi et al., 2018)] (*n* = 4)
	Increased WMH in CC [AD (Yoshita et al., 2006; Birdsill et al., 2014; Gaubert et al., 2021)] (*n* = 3)
	Increased WMH in CR [AD (Birdsill et al., 2014; Gaubert et al., 2021)] (*n* = 2)
	Increased PWMH [AD (Yoshita et al., 2006; Saka et al., 2007; Kim et al., 2011; Damulina et al., 2019; Graff-Radford et al., 2019; Weaver et al., 2019), MCI (Yoshita et al., 2006; Smith et al., 2011), PD (Grey et al., 2022)] (*n* = 9)
	Increased DWMH [AD (Stenset et al., 2008), MCI (Wu et al., 2019)] (*n* = 2)
	Increased WMH in ATR [AD (Torso et al., 2015)] (*n* = 1)
Other diseases	Increased DWMH [PDis (Bae et al., 2010), CAA (Charidimou et al., 2016)] (*n* = 2)
	Increased PWMH [HA (Charidimou et al., 2016)] (*n* = 1)
	Increased juxtacortical WMH [CAA (Phuah et al., 2022)] (*n* = 1)
	Increased frontal WMH [PDis (Bae et al., 2010), NDCS (McGuire et al., 2013), DM2 (Phuah et al., 2022), LC and BM (Quattrocchi et al., 2014)] (*n* = 4)
	Increased WMH in SLF [artd (Biesbroek et al., 2013)] (*n* = 1)
	Increased WMH in ATR [artd (Biesbroek et al., 2013)] (*n* = 1)

ACA, anterior cerebral artery; AD, Alzheimer’s disease; ATR, anterior thalamic radiation; artd, arterial disease; BM, brain metastases; CAA, cerebral amyloid angiopathy; CADASIL, cerebral autosomal dominant arteriopathy with subcortical infarcts and leukoencephalopathy; CC, corpus callosum; CR, corona radiata; HA, hypertensive arteriopathy; HLD, hyperlipidemia; HTN, hypertension; LC, lung cancer; MCA, middle cerebral artery; MCI, mild cognitive impairment; NDCS, neurologic decompression sickness; PCA, posterior cerebral artery; PD, Parkinson’s disease; PDis; panic disorder; SLF, superior longitudinal fasciculus.

## 4. Discussion

White Matter Hyperintensities burden has been linked to various impairments, diseases, pathologies, as well as (cerebro)vascular risk and clinical factors. Subsequently, it has been used as a biomarker in multiple studies, often summarized as total WMH burden or load (Debette and Markus, 2010). However, WMHs demonstrate spatial distributions in the brain that are specific to diseases and other clinical and risk factors. Investigation of the topographical aspects of this marker of cerebral small vessel disease, rather than summarizing it as a single, volumetric measure, can therefore give new insights into the underlying pathophysiology and studied correlates of interest.

This systematic review of 41 studies gives new insights into the topography of WMHs as a biomarker and provides strong evidence for the importance and benefit of studying their spatial distribution. In general, we differentiate between qualitative and semi-/quantitative WMH characterization approaches. While qualitative approaches are easy to perform manually in smaller cohorts, other approaches, such as probabilistic segmentation methods, can be utilized to extend the analyses to bigger cohort sizes. Importantly, with the event of deep learning enabled pipelines for white matter hyperintensity segmentation, fully automated, quantitative characterization has become more prominent in the literature. The latter has the benefit of removing inter-rate variability within a study, however, comparability of segmentation between studies may not be given, if different algorithms are used, due to the high heterogeneity of performance (Kuijf et al., 2019).

For spatial stratification, we identified three general approaches to studying the spatial patterns of WMH. These included ROI, PWMH/DWMH, and voxel-wise stratification. While a generally accepted methodology on how to categorize WMH is lacking, the described relationships with risk factors and clinical correlates demonstrated consistent trends. In general, it is difficult to define such a gold-standard for spatial analyses, as both hypothesis (ROI) and hypothesis-free (voxel-wise) approaches have merit and depend on the specific research question that is being answered. An ROI approach may yield new insights and potentially simplify the analysis, however, the selection of ROI definition needs to be based on the underlying biological hypothesis. A voxel-wise approach provides a more granular topographical investigation through a data driven approach, however, the resulting interpretation of the results should consider the anatomical structure of the brain, and potentially relate its findings back to commonly used ROIs to facilitate the interpretation of the findings. Despite the different approaches and use of varying WMH quantification techniques, the discussed findings presented in this review agreed in the direction of the found association, suggesting the presence of a biologic signal.

We defined WMH in this review according to the STRIVE criteria (Wardlaw et al., 2013), i.e., hyperintense signal in the white matter of presumed vascular origin, assessed using FLAIR imaging. However, this systematic review covered populations with various diseases, including AD, DEM and PD, for which, at least in part, it is still unclear whether SVD is etiologically involved in the neurodegenerative processes or occurs coincidentally. For example, it has been suggested that SVD might precede Parkinsonism related pathology, (van der Holst et al., 2015) whereas WMH and amyloid accumulations might be independent, but additive processes (Roseborough et al., 2017). Nevertheless, it is established that WMH are highly prevalent in these neurodegenerative diseases and play a key role in cognitive impairment, warranting their investigation (Debette and Markus, 2010; METACOHORTS Consortium, 2016; Croall et al., 2017).

The studies included in this systematic review investigated spatial patterns of WMH in populations with different diseases. Spatial stratification was predominantly performed in studies investigating cognitive impairment (*n* = 23), identifying differences in spatial patterns of WMH in PWMH (*n* = 7; for AD and MCI), (Yoshita et al., 2006; Saka et al., 2007; Kim et al., 2011; Smith et al., 2011; Damulina et al., 2019; Graff-Radford et al., 2019; Weaver et al., 2019) as well as in the parietal (*n* = 5; for AD and MCI), (Tullberg et al., 2004; Ai et al., 2014; Brickman et al., 2015; Graff-Radford et al., 2019; Moscoso et al., 2020) posterior (*n* = 4; for AD and MCI), (Al-Janabi et al., 2018; Gaubert et al., 2021; Garnier-Crussard et al., 2022; Thu et al., 2022) and frontal (*n* = 3; for AD) (Tullberg et al., 2004; Graff-Radford et al., 2019; Moscoso et al., 2020) lobar regions. In this particular study population, spatial patterns of WMH appear across the entire brain. However, given the high overall global disease burden in these patient groups, as well as heterogeneity of confounding factors that are present and methodological approaches for characterizing WMH burden used, it is difficult to identify a consensus on distinct, disease specific spatial patterns.

Three studies identified distinctive spatial WMH patterns for different subtypes of SVD (Auer et al., 2001; Charidimou et al., 2016; Phuah et al., 2022). While juxtacortical WMH were associated with probable CAA, (Charidimou et al., 2016) deep (frontal) WMH were predominantly found in patients with risk factors for arteriosclerosis, (Phuah et al., 2022) and temporal WMH with CADASIL (Auer et al., 2001). Again, the methodological approaches differed across studies, hindering a direct comparison. Another three studies explored spatial patterns of WMH in patients with PD (Dalaker et al., 2009; Fang et al., 2021; Grey et al., 2022). While one of them did not identify spatial patterns specific to patients with PD, (Dalaker et al., 2009). Fang et al. (2021) found an increased WMH burden in the frontal lobe, and Grey et al. (2022) identified increased PWMH burden. The differences in these findings may be explained due to the variations in age and sex distribution of the study populations, as well as the different methodology used for characterizing WMH. Additional studies are needed for these and other common diseases, such as stroke, to fully explore the topography of the cerebrovascular disease burden. Future research may also consider elucidating differences, not only between patient cohorts compared to the general population, but between patient cohorts of various diseases to further highlight the applicability of spatially specific disease burden as biomarkers for early disease prognostication.

Epidemiologic research over the past decades has highlighted the importance of vascular risk factors in WMH (Grimaud et al., 1996; Wardlaw et al., 2015; Das et al., 2019). Overall, studies have identified spatial heterogeneity of WMHs related to risk factors and clinical correlates. Localized WMH burden was found to be positively correlated with vascular risk factors, such as HTN, alcohol consumption, and history of smoking, as well as clinical correlates, such as sex (see Table 2). The studies described in this review also demonstrated that patients with HTN mainly presented with an increase in DWMH burden, whereas smoking and alcohol consumption were linked to an increase in PWMH. This supports the underlying assumption that deep and periventricular WMH are different etiologies that are individually affected by different vascular risk factors.

While only a few studies specifically investigated sex differences, the research findings highlighted in this systematic review suggest that it plays an important role in the spatial distribution of WMH, with men showing higher WMH in periventricular and PCA regions and women with higher frontal burden. Sex differences in total WMH burden, as well as differences in women pre- and post-menopausal, have been shown previously in the literature (de Leeuw et al., 2001; van den Heuvel et al., 2004; Das et al., 2019; Lohner et al., 2022; Schirmer et al., 2023). These findings, with the above described spatial differences in disease burden, however, have not been fully explored, but should be accounted for in future studies.

Our systematic review further identified different spatial patterns of WMHs associated with poor overall cognitive outcomes. The results, however, are inconsistent and warrant further, systematic studies. Only a few studies (*n* = 3) investigated different cognitive domains, specifically looking into spatial patterns in relation to executive function and episodic memory. While there were no distinctive WMH patterns in studies of older participants who ranged from cognitively normal to demented, a study in middle-aged, cognitively normal participants found a distinct disease burden topography. Specifically, Brugulat-Serrat et al. (2020) showed that cognitively unimpaired, middle-aged adults with an increase in DWMH burden performed poorer in tasks relating to executive memory, whereas increased PWMH burden was related to poor episodic memory. In other studies in older adults, Tullberg et al. (2004) demonstrated that poor executive function and episodic memory was related to increased frontal WMH, whereas Smith et al. (2011) found that these were associated with increased PWMH. While these findings are not exclusive of one another, however, the use of different definitions for spatial stratification hinders a direct comparison. Both studies suggest, however, that this spatial distinction might be an early marker for cognitive impairment in younger people, where the overall WMH burden in the brain is still relatively low. Nonetheless, further studies across the lifespan are needed to fully examine the described association.

While research into spatial patterns of WMH is a rapidly developing field that first emerged around 20 years ago with most publications in the last decade, a commonly agreed terminology does not yet exist, which hinders the identification of related literature. While our research terms included a large variety of descriptive words for WMH and topology, it is possible that studies were missed due to the large variety of nomenclature. Efforts reflecting other standardization approaches, such as STRIVE, (Wardlaw et al., 2013) may be warranted to significantly enhance the development of the field. In this systematic review, the varying definitions for spatial WMH burden did not allow to pool sufficient data for additional meta-analysis. Future research and meta-analyses would also significantly benefit from a consensus for standard terminology and of image analysis methodology for spatial patterns analyses of WMH.

In this systematic review, we discussed the evidence in the selected literature for spatial WMH patterns. While the data of the included studies did not allow for a quantitative synthesis and risk assessment of bias of results, intrinsic biases of the discussed studies exist. Investigating spatially stratified disease burden necessitates large cohort sizes to account for the general heterogeneity of WMH presentation. Here, cohort sizes varied significantly, ranging from 32 to 882 patients/participants, with most studies having cohort sizes of *N* < 200 (median cohort size 191.5). While smaller cohorts may be sufficient to provide first insights into spatial patterns of WMH, larger cohort sizes are likely necessary to fully uncover the driving factors in observed WMH topography, its associated risk factors and clinical correlates. Moreover, studies significantly varied in populations under investigation, with a total of 16 different diagnoses, in addition to “control” groups, with a range of 1–15 studies per diagnosis. Studies including MCI patients were most prevalent. As described above, more disease specific evidence is required to remove uncertainties in the results that are present due to intrinsic biases in the study cohorts. Additionally, most studies included in this systematic review were conducted in older populations (median cohort age: 69.9 years), specifically unraveling spatial patterns of WMH with respect to AD, MCI and normal cognitive function. In general, WMH is most prominent in aging populations, as age is a key determinant of overall WMH burden, enabling easier automated quantification, where small errors in segmentation accuracy often only relate to minute variations in relative burden. Only a few studies investigated spatial patterns of WMH in younger participants, however, these investigations focused on rather specific study populations, e.g., presenting with panic disorders or pilots. The results presented here may therefore not generalize well to the general population. Studies are warranted to disentangle spatial effects of WMH in younger people and populations with lower WMH burden from the general population, as well as overall population studies which include a wide age range.

This systematic review provides an overview of the state of spatial patterns of WMHs across cohorts in adults. While in this review, we only extracted literature from a single database (PubMed), it provides a good starting point for the emerging studies. Here, we focused on adults, but WMH can emerge earlier in life and is often linked with disease state. Overall this systematic review has met its expectations and achieved its objectives.

## Author contributions

JB, VL, and MS: conceptualization, methodology, writing—original draft, and writing—review and editing. JB and MS: investigation. JB: formal analysis and visualization. MS: supervision. All authors had full access to all the data in the study and take responsibility for the integrity of the data and the accuracy of the data analysis.
